# Improving fidelity of implementation of self-administered pulse oximetry and remote patient monitoring in Honduras during the COVID-19 pandemic

**DOI:** 10.3389/fmed.2026.1721063

**Published:** 2026-07-01

**Authors:** Kathryn W. Roberts, Berta Alvarez, Omar Diaz, Michael de St. Aubin, Salomé Garnier, Saul Cruz, Lorenzo Pavon, Rachel See, Anthony D. So, Ligia Paina, Tara Kirk Sell, Shiony Midence, Angela Ochoa, Homer Mejía Santos, Jonatán Ochoa, Sogeiry Solis, Devan Dumas, Margaret Baldwin, C. Daniel Schnorr, Alcides Martinez, Avi J. Hakim, Eric Nilles

**Affiliations:** 1 Harvard Humanitarian Initiative, Harvard T.H. Chan School of Public Health, Cambridge, MA, United States; 2 Brigham and Women’s Hospital, Boston, MA, United States; 3 Johns Hopkins Bloomberg School of Public Health, Baltimore, MD, United States; 4 Secretaria de Salud de Honduras, Dirección General de Redes Integradas de Servicios de Salud, Tegucigalpa, Honduras; 5 Secretaria de Salud de Honduras, Unidad de Vigilancia de la Salud, Tegucigalpa, Honduras; 6 Centers for Disease Control and Prevention, Atlanta, GA, United States; 7 Harvard University Medical School, Boston, MA, United States

**Keywords:** COVID-19, Honduras, implementation fidelity, pulse oximetry, remote patient monitoring, telehealth

## Abstract

During the COVID-19 pandemic, Honduras’ Secretariat of Health was eager to understand whether self-administered pulse oximetry could improve clinical outcomes of high-risk COVID-19 patients. While these interventions show promise for expanding healthcare access in lower- and middle-income countries, evidence about contextual implementation fidelity and effectiveness remains limited. We conducted a retrospective fidelity of implementation analysis of phone-based patient monitoring with or without self-administered pulse oximetry in Tegucigalpa and Comayagüela, Honduras. Using Carroll’s Conceptual Framework for Implementation Fidelity, we analyzed adherence (content, coverage, frequency, duration), delivery quality, participant responsiveness, and implementation strategies through trial data, acceptability assessments, and implementation documentation. The intervention achieved high fidelity: 98.9% coverage (1,821/1,841 eligible patients), 97.7% daily monitoring call completion, and 97.7% pulse oximeter utilization. Implementation strategies included culturally-adapted education materials, communication styles tailored to participants, and multi-disciplinary team engagement. Participant satisfaction was 99.2%, with minimal withdrawal (0.3%) and loss to follow-up (2.0%). However, only 28% of participants sought additional care when referred for warning signs. Unexpected benefits included reported positive impacts on participants’ mental health during isolation. This analysis demonstrates that remote patient monitoring and self-administered pulse oximetry implementation can achieve high fidelity in a lower-middle-income setting, including during a health emergency and where such interventions are novel. This challenges concerns about technology access and adoption in resource-limited environments. Future research should focus on hybrid effectiveness-implementation trials and strategies to improve referral adherence. These findings suggest that expanding implementation of these approaches may enhance healthcare access during emergencies and routine circumstances.

## Introduction

In 2020, Honduras’ Secretariat of Health (SESAL) established COVID-19 triage centers to streamline care and manage the increasing patient load and embraced remote care delivery for high-risk, non-hospitalized patients, following guidelines developed by the World Health Organization (WHO) (1, 2). In April 2021, the WHO endorsed self-administered pulse oximetry to detect silent hypoxia. SESAL adapted the intervention to the context, including technology penetration, telehealth experience, ability to self-isolate, and household support (2–4). While remote patient monitoring and self-administered pulse oximetry have been evaluated in COVID-19 and other clinical contexts, evidence about what strategies support implementation fidelity was needed, particularly in lower- and middle-income country (LMIC). In response, SESAL and partners implemented a randomized trial and sub-studies to establish clinical impact, acceptability, and implementation fidelity in the Honduran and health emergency contexts (5–11).

Remote patient monitoring and self-administered pulse oximetry were not part of usual care in Honduras prior to study implementation. A review of Latin American telehealth practices implemented during the COVID-19 pandemic found that the approach increased patient access and reduced healthcare facility burden (12). A seven-country systematic review of remote patient monitoring during COVID-19 found that implementation fidelity of home-based care is largely reliant on patient willingness to engage in daily monitoring (13). Concerns about the use of remote management in LMICs tend to focus on cost, lack of access to technology, electricity, and mobile networks, and perceived lack of knowledge or skills to participate, especially among marginalized populations 8, 12–20.

Self-administered pulse oximetry by COVID-19 patients has been studied in various settings, though studies in LMIC remain limited. A 2022 systematic review concluded that self-administered pulse oximetry can enhance remote patient management when devices are available, accurate, and used correctly, although only 1 of 13 studies included was conducted in an LMIC (Brazil) (21, 22). Concerns have been raised about the feasibility of this approach in LMIC, primarily due to pulse oximeter cost and limited health and technological literacy (5). While pulse oximeters may need to be provided in resource-constrained settings, the approach could be cost effective if it reduces morbidity and mortality without admitting patients or conducting in-person monitoring.

Honduras’ complex social and political landscape shaped intervention and implementation design. Honduras is the most economically unequal country in Latin America, which can influence availability of space or financial buffer to isolate, socially distance, or afford preventive measures (23, 24). As a lower-middle-income country where 45.6% of the economy is informal, Honduras faces governmental instability and high violence. Despite free public sector healthcare access for 90% of residents, the healthcare system experiences challenges aligning care availability and quality with patient needs, due to gaps in funding, staffing, and commodities (25–33).

Implementation fidelity, the degree to which a program is delivered as intended, is essential to understand intervention effectiveness and replicability, yet is often inadequately assessed. Carroll’s Conceptual Framework for Implementation Fidelity (CFIF) provides a structured approach to examine adherence (content, coverage, frequency, duration) and potential moderators including implementation strategies, delivery quality, and participant responsiveness (34). This framework is valuable for examining implementation in resource-constrained emergency contexts where competing demands, limited infrastructure, and novel interventions could compromise fidelity (34, 35). CFIF allows systematic documentation of both what was achieved and what strategies enabled that achievement, generating actionable evidence for future implementation.

This retrospective fidelity analysis examines the implementation of remote patient monitoring with and without self-administered pulse oximetry in Tegucigalpa and Comayagüela Honduras, among people with COVID-19 at high risk of experiencing negative outcomes. The analysis uses the CFIF to assess adherence, implementation strategies, delivery quality, and participant responsiveness (34). Remote patient monitoring and related technologies may become vital tools for expanding healthcare access during emergencies and routine care; understanding implementation fidelity in LMIC settings is essential for informing future intervention design and scale-up.

## Materials and methods

We conducted a retrospective, observational study of the implementation fidelity of a cluster-randomized trial assessing phone-based remote patient monitoring with and without self-administered pulse oximetry for non-hospitalized COVID-19 patients at high risk for severe disease in Tegucigalpa and Comayagüela, Honduras. Relying on the CFIF, fidelity encompasses adherence (comprised of intervention content, coverage, frequency, and duration), while the relationship between the intervention and adherence may be moderated by intervention complexity, implementation strategies, delivery quality, and participant responsiveness (34). We conducted this analysis using existing data, including data from the parent trial; a mixed methods acceptability study; trial implementation notes; and the implementation handbook and standard operating procedures, which served as the benchmark against which fidelity was assessed. Detailed methods, procedures, and results from the parent trial and the acceptability sub-study are reported separately (36, 37).

### Intervention design and setting

The parent trial and interventions are described briefly to provide context, further detail can be found in the intervention impact manuscript (36). SESAL established COVID-19 triage centers in Tegucigalpa and Comayagüela, which were the primary public sector sites for COVID-19 diagnosis and care. SESAL- employed physicians were asked to refer patients for eligibility screening who: (1) were SARS-CoV-2 positive via rapid antigen test, and were (2) 60 + OR (3) 45 + with at least one specified condition; telephone ownership was not screened for prior to initial referral. Participants were enrolled between March 30, 2022 and January 24, 2023 by study-employed physicians at five COVID-19 triage centers managed by SESAL. Study-eligible patients (1) self-presented to a SESAL-managed triage center, and were (2) SARS-CoV-2 positive via rapid antigen test, (3) symptomatic for SARS-CoV-2, (3) 60 + or 45–59 with CDC-defined high-risk conditions (diabetes, hypertension, obesity, chronic lung disease, immunosuppression, cardiovascular disease, chronic kidney disease, chronic liver disease, or pregnancy), (4) discharged home from a triage center, and (5) had access to a working telephone during the enrollment period (38). Of 1,860 patients assessed for eligibility, 39 were excluded (2.0%): 19 did not qualify, 19 declined to enroll, and 1 was enrolled twice. The final study population comprised 1,821 participants, 924 in the daily monitoring only arm, and 897 in the daily monitoring and pulse oximetry arm (Supplementary material S1).

At enrollment, participants provided informed consent, were administered questionnaires, and received health education. Participants were randomized to receive daily, telephone-based remote monitoring, with or without a pulse oximeter. Monitoring was conducted for 10 days from symptom onset, unless participants reported ongoing fever, in which case monitoring continued until the participant was fever-free for 24 h. Those in the pulse oximetry group were given a CDC-approved pulse oximeter (MedLine Soft Touch Fingertip Pulse Oximeter (Northfield, IL) or CuraPlex Fingertip Pulse Oximeter (Dublin, OH)), usage instructions, and a diary for SpO_2_ readings (Supplementary material S2). During monitoring calls, study-employed nurses assessed participant warning signs; if any were identified, including SpO_2_ ≤ 94%, participants were referred for in-person evaluation. Participants were asked to return pulse oximeters to triage centers for sterilization using alcohol and re-distribution. To encourage returns, participants were offered ~$10 USD to cover transportation expenses. The trial’s primary outcome was reduced mortality among participants using pulse oximeters, which is reported in an independent manuscript (36).

### Acceptability study

The acceptability sub-study and methods are summarized here, further detail is available in a separate manuscript (37). Acceptability was assessed using domains described by Sekhon et al. (39, 40). An exploratory sequential mixed methods design was chosen to provide depth and breadth to the investigation. The integration of quantitative and qualitative methods occurred during study design, analysis, interpretation, and reporting, as described by Fetters et al. (41). During the trial, 1,767 participants (900 from the daily monitoring only arm and 868 from the daily monitoring with pulse oximetry arm) responded to quantitative and open-ended acceptability questions, asked on their final day of enrollment, allowing researchers to draw conclusions about the study population, while open-ended questions permitted participants to express opinions. In months nine and ten of the trial, 19 SESAL healthcare providers (three physician administrators and 16 physicians) and 15 study staff (eight physicians and seven nurses) were purposively selected to respond to a preliminary, anonymous, computer-aided self-interview (CASI) including quantitative and open-ended questions exploring study perceptions, given limited information about their perspectives (39, 42). In-depth interviews were conducted with a sub-set of the CASI respondents, randomly chosen from among the CASI participants who volunteered to be interviewed. Interviews were conducted by senior study staff with eight SESAL staff and eight study staff after trial conclusion, in January – March 2023, which enabled examination of rationale for perspectives expressed in the CASI.

### Fidelity evaluation

**Figure 1:**
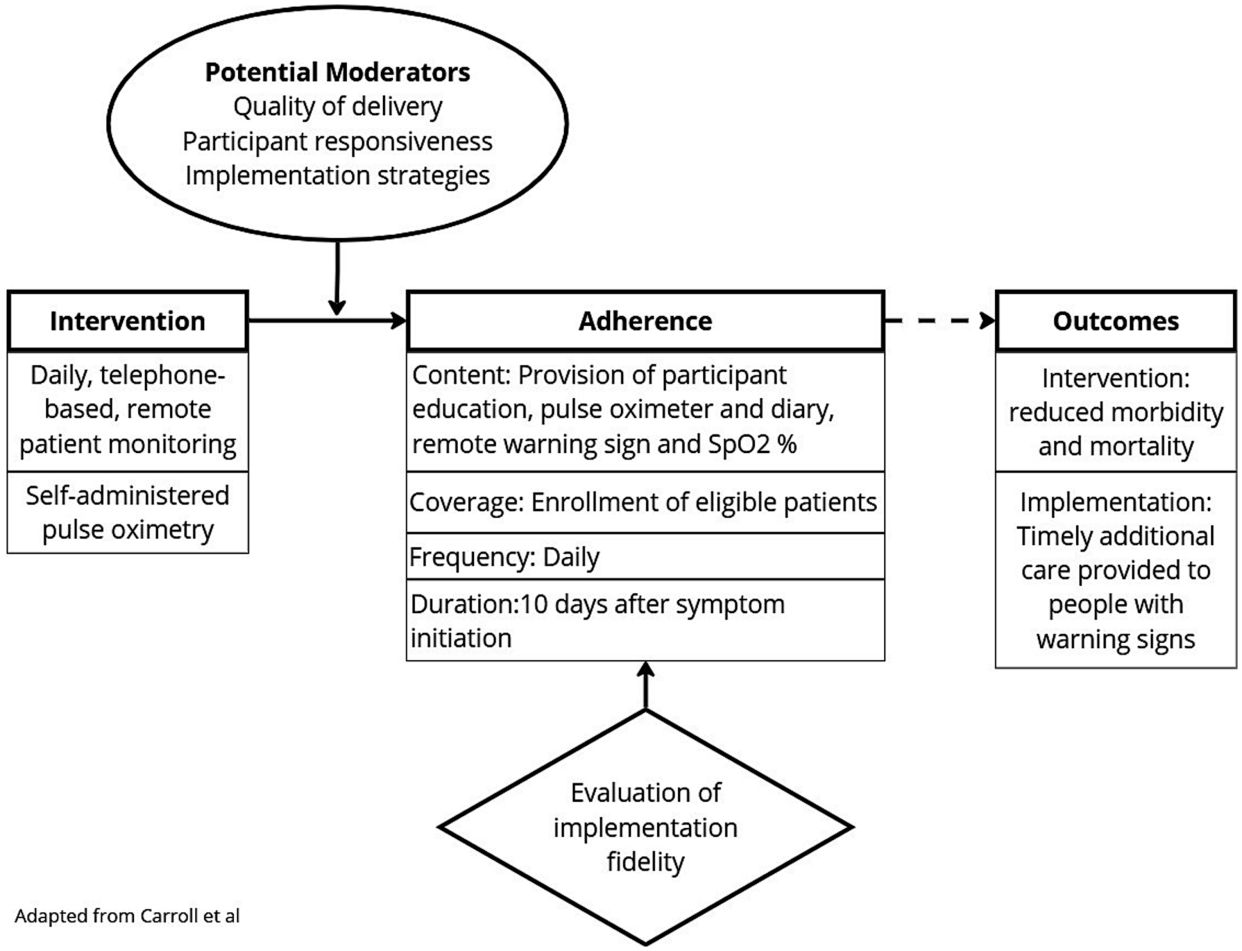
Adapted conceptual framework for implementation fidelity (CFIF) (34). Sp0_2 –_ Peripheral oxygen saturation, or blood oxygen.

In collaboration with SESAL, the study management team tailored strategies to enhance implementation, delivery quality, and participant responsiveness to optimize fidelity. The adapted CFIF (Figure 1) was used to develop the fidelity evaluation approach described below, where each of the elements of the CFIF - adherence, potential moderators (quality of delivery and participant responsiveness), and strategies employed to strengthen adherence, are evaluated using multiple outcome measures from several sources to allow comparison and, potentially, triangulation.

Data sources employed in the fidelity evaluation included: related data from the parent trial and the acceptability sub-study, described above, the trial handbook containing implementation instructions and standard operating procedures served as the benchmark against which fidelity was assessed, and field supervision and implementation notes from the study team. Each component of fidelity is assessed using a combination of these data sources. We report fidelity components separately rather than as a composite metric, as no validated fidelity composite measure exists to our knowledge and component-level reporting provides more actionable implementation guidance.

Below we describe the data sources used to assess each fidelity component.

We examine the components of adherence using data collected during the parent trial and information from the implementation handbook (Table 1). Adherence was operationalized as the extent to which the intervention was delivered according to the handbook.

**Table 1:** Intervention fidelity outcome measures.

**Fidelity component**	**Fidelity outcome measures**
Adherence	Proportion of intervention content delivered as intended including: COVID-19 education, study supplies delivery at enrollment, screening for warning signs and low SpO_2_ (≤94%), and referral for additional care when identified^1,3^
	Coverage of the target population, i.e., proportion of eligible patients enrolled^1^
	Frequency of remote patient monitoring calls made by study staff versus frequency intended^1^
	Duration of participant enrollment in the intervention versus enrollment intended^1^

Data sources: (1) quantitative trial data, (2) implementation handbook. Abbreviations: Sp0_2 –_ Peripheral oxygen saturation, or blood oxygen.

Potential moderators of the relationship between implementation and adherence include implementation strategies, delivery quality, and participant responsiveness, which are interconnected (Table 2).

**Table 2:** Outcomes of quality of delivery and participant responsiveness as potential moderators of the relationship between the intervention and adherence.

**Potential moderators**	**Outcomes**
Quality of delivery	Study physician and nurse preparedness, enthusiasm, and willingness and ability to answer questions^2,3,4^
Participant responsiveness	Withdrawal and loss-to-follow-up rates among participants^1^
	Proportion of participants with pulse oximeters that report using them^1^
	Proportion of participants referred for additional care who sought it out^1^
	Pulse oximeter return rate^1^

Data sources: (1) quantitative trial data, (2) acceptability data, (3) implementation handbook, (4) trial implementation notes.

Implementation strategies aim to improve delivery quality and participant responsiveness; delivery quality may influence responsiveness. Participant responsiveness was measured at each stage of the intervention where participants could choose to disengage. Implementation strategies were developed to improve adherence, delivery quality, and participant responsiveness (Table 3).

**Table 3:** Implementation strategies employed to enhance quality of delivery and participant responsiveness.

**Potential moderators**	**Strategies**
Team composition	Engagement of a multidisciplinary team to lead intervention adaptation and implementation^4^
Preparation and engagement	Study staff training and intervention piloting^3,4^
	Ongoing triage center staff engagement^3,4^
Adaptation to the participant population	Questionnaire piloting with the target population^3,4^
	Participant education about COVID-19 and pulse oximetry adapted to the target population^2,3^
Operations priorities	Intervention availability 12/7 to meet target population demand^1,3,4^
	Strong supply chain focus to ensure intervention delivery at triage centers^1,3,4^
Technology for communication and data	Technology-driven communication strategies to connect with participants and remote teams and ensure accurate intervention implementation^3,4^
	Tablet-based data collection to enable population of a dashboard for near real-time staff and implementation oversight^1,3,4^
	What’s App messages prior to remote patient monitoring calls^3,4^
Supporting participants	Providing participants with COVID-19 transmission prevention kits containing N-95 masks, surgical masks, bar soap, and for half of participants, hand sanitizer as well
	Trust building before and during remote patient monitoring calls^2,3,4^
	Transportation reimbursement to support pulse oximeter return to triage centers^1^

Data sources: (1) quantitative trial data, (2) acceptability data, (3) implementation handbook, (4) trial implementation notes.

Many strategies targeted multiple areas of fidelity simultaneously. For example, piloting questionnaires with the target population helped ensure questions were easy to ask, supporting adherence, and easy to answer, promoting responsiveness and continued participation. Additionally, questionnaire piloting aimed to improve delivery quality by ensuring study staff had practice and received feedback from supervisors.

### Analysis

Quantitative analysis of parent trial and acceptability data involved the calculation of descriptive statistics, reported as frequencies and percentages. Enrollment refusal was analyzed using logistic regression assessing age and gender. Education data were not available for patients who declined prior to providing consent. Reasons given for refusal to enroll were analyzed qualitatively and categorized. Quantitative trial disenrollment and loss-to-follow-up data were analyzed using logistic regression with difference assessed by sociodemographic variables, and intervention assignment. For the association between phone ownership status and withdrawal, Fisher’s exact test was used due to zero events in the borrowed phone group. Exploratory logistic regression analysis was conducted to examine the relationship between participant age and phone ownership patterns. The outcome variable was phone ownership (owned phone vs. borrowed phone from household or non-household member). Age was modeled as a categorical variable in 5-year increments, with 45–50 years as the reference group. This exploratory analysis was conducted post-hoc to characterize technology access patterns in the study population and was not pre-specified as a study objective. Further details, including study size calculations, can be found in the intervention impact manuscript (36). Missing data were minimal in the parent trial, with 5 participants withdrawing and 37 lost to follow-up, as described in the results. Participants were considered lost to follow-up after three consecutive days of being unreachable. The trial employed an intention-to-treat approach, whereby all enrolled participants were included in analysis. Quantitative data were analyzed in STATA/17.0, 2021 and R 4.2.2, 2022 and reported according to CONSORT guidelines (43, 44). We report unadjusted odds ratios with 95% confidence intervals.

Responses to open-ended questions collected during in-depth interviews as part of the acceptability sub-study were thematically categorized and summarized separately from other qualitative data collected. In-depth interviews were analyzed using deductive framework analysis grounded in Sekhon et al’s work with space for inductive code generation based on discussion between the two coders and framework functionality in relation to the data. A codebook was developed to ensure transparency and coding alignment. Coders used regular discussion and collaborative memoing, where qualitative researchers record thoughts, insights, and initial interpretations in shared documents, to ensure reflexivity (45). Each coder reviewed all transcripts for framework and code development. Next, each coded two transcripts, reviewed the other’s work, discussed disparities in code application, and updated code definitions. Then, each led coding on half the remaining transcripts, which the second coder then reviewed. Qualitative data were organized in Dedoose and Microsoft Excel (46, 47). All data were collected in Spanish and all textual data analyzed in Spanish, with results translated by K. Roberts. A document review was conducted of the implementation handbook and implementation notes taken by study staff, focusing on alignment between the handbook and actual practices.

Potential biases varied by data source. Trial data could reflect selection bias, whereby only people who self-presented to the triage centers were eligible for enrollment. Participant acceptability data were subject to selection bias, as we used purposive sampling, and social desirability bias as study staff administered surveys; we partially addressed this through the use of CASI. Healthcare provider interviews risked social desirability bias, as senior study staff conducted them and recall bias, as they occurred post-trial. Implementation documentation may reflect observer bias as the team documented their own processes; but given that supervisors conducted the assessments, they were motivated to identify discrepancies.

### Ethical approval

Institutional Review Board (IRB) review was received from the Massachusetts General Brigham (2021P001143) and the Autonomous University of Honduras (00003070). Secondary data analysis was approved by the Johns Hopkins Bloomberg School of Public Health (24586).

## Results

Implementation fidelity results related to adherence, quality of delivery, participant responsiveness, and implementation strategies are presented below.

### Adherence

Adherence was assessed by examining intervention coverage, content, frequency, and duration. The intervention achieved 98.9% coverage, enrolling 1,821 out of 1,841 eligible patients (Table 4). Among those not enrolled (*n* = 39), 19 did not qualify, 19 declined participation, and one was enrolled twice (Supplementary material S2).

**Table 4:** Adherence results.

**Fidelity outcome measures**	**Results** Numerator/Denominator (%)
Content: Number of participants to whom intervention content delivered as intended / total number intended per protocol	
COVID-19 education	1,821/1821 (100%)
Study supplies delivery at enrollment	1,821/1821 (100%)
Screening for warning signs and low SpO_2_ (≤94%)	12,307/12,307 (100%)
Referral for additional care when SpO_2_ below ≤94% identified	43/43 (100%)
Referral for additional care when warning signs identified	108/116 (93.1%)
Coverage of the target population: Number of eligible patients enrolled / total patients eligible to participate^1^	1,821/1,841 (98.9%)
Frequency: Number of completed remote patient monitoring calls made by study staff / Number of calls attempted^1^	12,307/12,602 (97.7%)
Duration: Intended: Mean enrollment days for all participant; Actual = (mean days post symptom onset participants are enrolled) + (mean days enrolled)	Intended: 10 days post symptom onset Actual: 10.3 days (2.9 days + 7.4 days)

Data sources: (1) trial data, (2) implementation handbook.

Intervention content was delivered consistently. All 1,821 participants received COVID-19 education and appropriate study supplies at enrollment, with no reported stockouts. Screening for warning signs and low SpO_2_ (≤94%) was conducted during each remote monitoring call where a patient was reached (*n* = 12,307); the participant was reached on 97.7% of all calls (12,307/12,602). All participants reporting an SpO_2_ below ≤94% during a monitoring call (*n* = 43) were referred. Of the 116 remote monitoring calls with reported warning signs, healthcare staff failed to refer participants on eight occasions (6.9%). Reasons participants were not referred included: one participant seeking care outside the triage system, one sought additional care the day prior, and another reported the warning sign chronically, the remainder did not provide explanations. In response to non-referrals, staff received additional training to emphasize mandatory referrals, and form prompts and constraints were added to ensure compliance.

Frequency of remote patient monitoring calls aligned with expectations, each participant was called daily, and if not reached, called up to two more times that day. 97.7% of daily monitoring calls were completed (*n* = 12,307). Seventy-three calls were made to people who could not be contacted and later classified as lost to follow-up, defined as not answering the monitoring call on three consecutive days. Due to an implementation error, two enrolled participants were called 10 times each without being reached; they were subsequently classified as lost to follow-up. Expected intervention enrollment duration was 10 days post-onset of COVID-19 symptoms; enrollment occurred a mean of 2.9 days (SD 1.5) after symptom onset and participation duration mean was 7.4 days (SD 1.4).

### Moderating factors

#### Quality of delivery

Quality of delivery was assessed through staff supervision and participant feedback. Supervision demonstrated that staff adapted their educational and communication approaches based on participants’ reading and health literacy levels, and understanding of COVID-19 (Table 5).

**Table 5:** Quality of delivery results.

**Quality of delivery outcome measures**	**Results** Numerator/Denominator (%)
Participant-rated satisfaction with the intervention they received (participants self-describing as satisfied or very satisfied with the intervention they received/total question respondents)	1,740/1,754 (99.2%)
Participant willingness to participate in the intervention again if they contracted COVID-19 (total participants willing to participate in the intervention again / total question respondents)	1,667/1,767 (94.3%)
Study physician and nurse preparedness, enthusiasm, willingness, and ability to answer questions^2,3,4^	Study notes of triage center observations

Data sources: (1) Trial data (2) Acceptability data (3) Implementation handbook (4) Implementation notes.

According to participant responses to acceptability questions, 99.2% (*n* = 1,740) of respondents expressed satisfaction with the intervention, and 94.3% (*n* = 1,667) were willing to participate again if they contracted COVID-19. A small portion, 0.6% (*n* = 11), were unsure, while 5.0% (*n* = 89) indicated they would not participate again.

Senior study staff observed study physicians and nurses consistently using kind and empathetic communication techniques, such as greeting patients, speaking slowly and clearly, and answering participant questions, which was emphasized during training and in the implementation manual. They dedicated significant time to supporting participants and incorporating family members into education and monitoring, when appropriate.

#### Participant responsiveness

Participant responsiveness was assessed through study withdrawal and loss to follow-up, pulse oximeter use, responsiveness to referrals for additional care, and pulse oximeter recapture rate (Table 6).

**Table 6:** Participant responsiveness results.

**Participant responsiveness outcome measures**	**Results** Numerator/Denominator (%)
Participants that withdraw from the study/Total participants enrolled^1^	5/1,821 (0.3%)
Participants lost-to-follow-up/ Total participants enrolled^1^	37/1,821 (2.0%)
Participants with pulse oximeters that report using them/Participants that received pulse oximeters^1^	850/870 (97.7%)
Participants referred for additional care who sought it out/Participants referred for additional care^1^	28/100 (28%)
Pulse oximeters returned/Pulse oximeters dispensed ^1^	614/897 (68.5%)

Data sources: (1) trial data.

Of 1,860 patients assessed for eligibility, 19 (1.02%) declined to enroll. We assessed potential predictors of enrollment refusal using univariate logistic regression. Neither age nor gender were significantly associated with enrollment refusal (age: OR 0.98, 95% CI 0.94–1.02, *p* = 0.362; gender: *p* = 0.007). All 19 participants who declined provided reasons for refusal, which included: perceived lack of necessity for monitoring (*n* = 5, 26%), unwillingness to commit to phone-based follow-up (*n* = 5, 26%), desire to return home to rest (*n* = 2, 11%), already recovering or late in illness course (*n* = 2, 11%), and various individual reasons including privacy concerns, work constraints, existing healthcare coverage, and preference for device provision without monitoring (*n* = 5, 26%). Notably, no participants cited lack of phone access as a reason for declining enrollment; lack of a working telephone was recorded as a reason for ineligibility for one patient.

Of 1,821 participants enrolled, 0.3% (*n* = 5) withdrew, and 2.0% (*n* = 37) were lost to follow-up. We assessed potential predictors of study withdrawal and loss to follow-up using univariate logistic regression. None of the examined variables were significantly associated with withdrawal (age: OR 1.01, 95% CI 0.93–1.10, *p* = 0.867; gender male vs. female: OR 2.34, 95% CI 0.39–14.03, *p* = 0.353; education secondary vs. university: OR 0.24, 95% CI 0.03–2.18, *p* = 0.206) or loss to follow-up (age: OR 1.00, 95% CI 0.97–1.02, *p* = 0.733; gender: *p* = 0.250; education: *p* = 0.320; intervention assignment: OR 1.59, 95% CI 0.89–2.86, *p* = 0.116). All five withdrawals occurred among participants randomized to the pulse oximetry arm; no participants in the monitoring-only arm withdrew. Those who withdrew described phone calls as bothersome.

Participant engagement was evident in the high rates of successful remote patient monitoring calls, described earlier. Among participants in the pulse oximetry arm, 97.7% reported using the device as directed; 2.3% (*n* = 20) reported not using it.

Participant responsiveness to referrals for additional care after warning signs or an SpO_2_ ≤ 94% was lower than expected. Overall, 100 participants were referred for follow-up during 116 calls where warning signs were reported; participants were re-referred on subsequent calls if symptoms persisted. Of these, 52 reported seeking care or the outcome of their referral remained unknown. During chart review to determine final outcomes, study staff followed up with participants when no chart or other evidence of return for care could be found. During follow-up calls, 24 participants revised their initial responses, indicating that they had not sought additional care. Ultimately, 28% (*n* = 28) of participants sought care after referral, with 27 attending SESAL or IHSS health facilities and one attending a private health facility.

Pulse oximeter recapture aimed to enhance the intervention’s cost-effectiveness. Participants were asked to return devices to be sterilized and redistributed. To encourage returns, participants were offered ~$10 USD to cover transportation expenses. The study purchased 500 pulse oximeters; because returned devices were cleaned and reused, these supplied all 897 participants in the pulse oximetry arm. Of the 897 devices dispensed, 614 were returned, a recapture rate of 68.5%.

#### Implementation strategies

##### Team composition

The multi-disciplinary study team consisted of public health practitioners, medical doctors, epidemiologists, program managers, and a microbiologist. The team worked closely with key stakeholders from national, departmental, and city-level SESAL offices, as well as COVID-19 triage centers. In-person and virtual stakeholder meetings were held regularly to ensure consistent communication and coordination.

##### Preparation and engagement

Study staff underwent 5 days of training and intervention piloting, led by SESAL and study staff. Training included problem-solving exercises and role-playing participant interactions to refine communication techniques and ensure high intervention quality. Job aids outlining intervention participation criteria were posted in exam rooms to support triage physicians, and paper invitations were given to patients to encourage them to visit the study enrollment site within the triage center after referral by a triage center physician. Only 1.0% (*n* = 19) of referrals were erroneous.

##### Adaptation to the participant population

Piloting the study’s questionnaires with potential participants helped ensure questions were easy to understand. One notable issue was a question about whether participants felt their health had returned to its pre-COVID baseline, which led to confusion, with participants often reporting that they felt better after versus before their COVID-19 illness, despite reporting persistent symptoms. As a result, this question was excluded from analysis.

During enrollment, study physicians delivered education about COVID-19 transmission, warning signs, and pulse oximeter use. Staff engaged family members who accompanied participants to reinforce study instructions at home. A printed SpO_2_ diary with visual and written instructions, along with an in-person demonstration, were provided to enable thrice-daily readings, recording, and reporting them during remote monitoring calls.

##### Operations priorities

A staggered rollout approach was used for implementation. On the first day, the study team and SESAL collaborators attended a single triage center to practice skills, observe operations, and provide feedback to ensure consistent and quality implementation. Additional study sites were added over 1 week. Once fully operational, study physicians were stationed at COVID-19 screening sites 12 h a day, 7 days a week, matching hours of highest patient volume. Triage centers were open 24 h a day, and participants were instructed at enrollment to seek care immediately if warning signs arose, including outside of monitoring calls. Two triage centers closed during the study, and one enrollment site was added at the Social Security Hospital. Enrollment schedules were adjusted when COVID-19 rates declined between waves, reducing staffing and expenses to allow intervention extension.

Ensuring daily supplies for enrollment sites was a complex and essential task. During implementation every participant received the appropriate supplies at the time they were enrolled, thanks to a study coordinator who managed a central commodities store, inventory at remote enrollment sites, and could quickly provide additional supplies when needed, supported by a dedicated study vehicle and driver.

##### Technology for communication and data

Phone-based monitoring was effective throughout the trial. Early concerns about phone ownership among participants were allayed due to the urban setting and feedback from study staff. Among enrolled participants, 92.9% (*n* = 1,692) owned a phone, 6.8% (*n* = 124) borrowed one from a household member, and 0.3% (*n* = 5) borrowed from someone outside their household. Of the 19 patients who did not qualify for enrollment, one was unable to access a telephone during the enrollment period. In exploratory analysis, phone ownership declined significantly with age: compared to participants aged 45 to 50 years, those aged 85 and older had markedly higher odds of using a borrowed phone to participate (OR 52.99, 95% CI 20.81 to 134.96). Phone ownership status was not associated with study withdrawal; none of the 129 participants using a borrowed phone withdrew, compared to 5 of 1,692 using an owned phone (0% vs. 0.30%, *p* = 0.536, Fisher’s exact test).

WhatsApp, which is highly accessible and included with pre-paid phone credits in Honduras, was an effective communication tool, supporting smooth operations and used in participant communication. Daily reminders for staff addressed enrollment logistics, staffing and supervision, changes at triage centers, supply management, and clarification of enrollment criteria. Initially, when participants were referred for additional care, triage center staff had no way to know the patient was part of the study, or which warning sign had triggered the referral. In response, digital referral slips were introduced, identifying the participant as part of the study and listing the referral reason. These digital slips were sent to participants via WhatsApp.

Data were collected using Kobo Collect on tablets and uploaded daily to a secure cloud-based server via secure Wi-Fi. Automated spreadsheets tracked participants and daily call lists, while a Power BI dashboard facilitated supervision and information sharing among the study team, implementing team, and SESAL staff. Regular data reviews helped monitor protocol adherence, including enrollment, participant inclusion criteria, delivery of intervention components, enrollment days, and referrals after warning signs were reported. As protocol deviations were identified, pop-up warnings and constraints were added to electronic forms to reinforce compliance.

##### Participant support

The provision of COVID-19 transmission prevention kits, which contained N-95 and surgical masks, bar soap, and alcohol-based hand rub (distributed to 50% of participants), may have enhanced study enrollment. The timing of the study coincided with a period of decreased COVID-19 concern, suggesting that participants who actively sought diagnosis were health-conscious and thus may have been particularly receptive to receiving preventive supplies as a study incentive.

Establishing participant trust was essential to encourage continued engagement and maintain quality delivery. This process began by adhering to familiar Honduran greeting styles and communication patterns, allowing sufficient time for participant interaction to ensure understanding and a trusting relationship. As mentioned previously, sending WhatsApp messages to study participants prior to the monitoring phone calls further strengthened trust by reassuring participants of the legitimacy of the calls. Ensuring that all aspects of the intervention were delivered as promised—from providing supplies during enrollment to initiating daily monitoring calls at the participant’s preferred time and issuing referral slips—was key to building confidence in the study and fostering engagement.

At enrollment, participants were asked to return pulse oximeters after participation. Study nurses reminded participants on their final day of enrollment and followed up through additional calls, if time allowed. Messaging encouraged participants to return pulse oximeters to aid the study and to benefit their fellow Hondurans. Upon return, participants received a phone credit valued at approximately $10 USD to cover transportation costs.

### Adaptation in line with emerging evidence

From study conceptualization in 2021 to conclusion January 2023, key changes occurred. Shortly after study inception, the CDC updated the pre-existing conditions linked to COVID-19 risk (when five participants were enrolled); thereafter hypertension was added as a qualifying condition. Additionally, the minimum enrollment age was lowered from 50 to 45 years for those with comorbidities, and from 65 to 60 years for those without. After emerging evidence found that pulse oximeters overestimate SpO_2_ levels by 1.1–1.2% in people with darker skin, the study team emphasized mandatory referral after warning sign reports, including if SpO_2_ was >94% (48).

### Harms and unintended effects

No unintended harms from study participation or interventions were identified. A notable unintended positive effect was widely reported: participants described meaningful improvements in their mental and emotional health during COVID-19 illness and isolation, attributing this to the daily contact, care, and validation provided during monitoring calls. This was reported consistently enough by participants, study nurses, and SESAL providers that several of the latter believed it was a study objective. SESAL providers also reported that the intervention may have reduced triage center burden by decreasing unnecessary care visits, though corroborating data are unavailable. As no validated mental health instrument was used, we cannot characterize the magnitude or duration of this effect.

## Discussion

This analysis demonstrated that remote patient monitoring and self-administered pulse oximetry achieved high implementation fidelity in a lower-middle income urban setting during a health emergency, with near-universal coverage (98.9%), monitoring call completion (97.7%), and device use (97.7%). However, a critical implementation gap emerged in referral adherence (28%), highlighting that even if the core intervention is delivered with high fidelity, attention must be paid to enabling ongoing participation at all stages of the intervention.

Common concerns about implementing remote patient monitoring in LMIC settings focus on electricity, mobile networks, technology literacy, equitable access, and cost (8, 12–20). Our findings from this urban setting with high mobile phone penetration suggest that these barriers are not universal in LMIC settings. Among 1,860 patients assessed for eligibility, only one was ineligible for enrollment due to lack of phone access (0.05%), and participants successfully used telephones and pulse oximeters despite minimal prior experience with these technologies. However, technology access was not uniform. We identified a significant age-related disparity in phone ownership, with the oldest participants (85 + years) far more likely to borrow a phone than participants 45–50 years old (OR 52.99 [CI 20.81–134.96]). Direct analysis of the association between phone ownership status and study retention revealed that none of the 129 participants using borrowed phones withdrew from the study, compared to 5 of 1,692 participants using owned phones (0% vs. 0.30%, *p* = 0.536), providing empirical evidence that phone borrowing did not negatively affect retention. Nevertheless, unmeasured selection bias may have occurred if eligible older adults without phone access never presented for enrollment, or if triage center physicians pre-screened for phone ownership, against study guidance. These findings have important implications for equitable scale-up. Implementation teams should proactively assist participants in identifying phone access within their household or social network, as occurred during this trial. For individuals who cannot access any phone, alternative delivery mechanisms such as community health worker home visits will be essential to prevent systematic exclusion of the most vulnerable populations.

Participant responsiveness exceeded that reported in other programs. A seven-country systematic review of remote monitoring during COVID-19 found patient engagement was a frequent fidelity gap, and a similar UK program had very low intervention enrollment (1.17%) among eligible patients (13, 49). This high participant responsiveness could be context- or population-specific, such as greater willingness to receive calls due to isolation or the older population, warranting further investigation with other populations and settings.

Several factors appear to have supported high operational fidelity. Multi-disciplinary team composition enabled comprehensive problem-solving across clinical, public health, and logistical domains, preventing single-discipline blind spots. Staggered rollout with continuous feedback loops allowed real-time problem identification and correction before issues became systemic. When staff failed to refer eight participants despite warning signs, immediate retraining and form modifications prevented recurrence. Dedicated operational roles (e.g., supply coordinator, data manager) ensured enrollment sites never experienced stockouts and staff could focus on quality patient interactions. This urban infrastructure provided critical advantages: reliable cellular networks enabled consistent contact, and proximity to triage centers made device returns feasible. Finally, family member involvement, although not measured quantitatively, likely contributed to the high call completion and device use rates we observed, particularly for older participants and those using borrowed phones. In multigenerational households typical of Honduras, family members can support call completion, daily SpO2 measurement, symptom recognition, and care-seeking decisions.

Quality of delivery likely influenced patient responsiveness at multiple touchpoints (34). Responsiveness was crucial at each intervention step, from enrollment, to phone calls, describing warning signs, using pulse oximeters, acting on referrals, and returning pulse oximeters. Implementation strategies that may have enhanced delivery quality included: text messages before calls, culturally appropriate conversation before health assessments, and other small modifications based on participant feedback (50). These approaches appear to have built trust, which has been shown to promote health system engagement and improve clinical outcomes (51–53). Many of these strategies emerged through iterative engagement with the participant population during design and implementation rather than being prescribed by researchers. WhatsApp messaging, although introduced as an operational tool, likely functioned as a meaningful component of the intervention by pre-announcing calls, reinforcing trust, and enabling digital referral slips.

Low referral adherence represents the study’s most significant implementation gap. We found no comparable benchmarks for referral adherence following remote patient monitoring during COVID-19, making direct comparison difficult. Notably, while several studies have evaluated remote patient monitoring with pulse oximetry during COVID-19, none reported referral adherence rates despite referral being a key component of these interventions (13, 21, 49, 54, 55). This gap in the literature suggests referral adherence may be under-recognized as an implementation challenge, or at the very least bears measurement and reporting, in remote monitoring programs. While substantial literature documents referral adherence barriers in LMIC health systems, the pandemic emergency context introduced distinct implementation challenges that limit comparability. Mandated isolation, fear of nosocomial COVID-19 transmission and re-infection, and the asynchronous nature of remote assessment, where participants often felt improved hours after a monitoring call identified warning signs, created barriers fundamentally different from traditional referral pathways.

Healthcare provider interviews identified three intersecting barriers: participants feeling improved (reducing perceived urgency), anticipating long wait times at triage centers (opportunity cost of time), and lacking transportation funds (economic barrier). These align with well-documented access barriers in LMIC health systems. The contrast between high monitoring call completion and low referral adherence suggests the primary barrier may be accessibility constraints at the referral stage rather than patient disengagement. Interestingly, device return rates were substantially higher than referral adherence despite both requiring triage center visits. Possible explanations include timing (device return occurred post-recovery) and the time required (dropping off a pulse oximeter versus waiting in line to see a provider); the additional drivers of device return are discussed below.

Addressing this gap will be critical for future implementation. Strategies to explore include: immediate mobile money transfers for transportation costs related to referrals; digital referral slips guaranteeing priority triage to reduce wait times; telemedicine consultations before requiring in-person visits for those with mild symptoms; and community health worker partnerships for in-person assessments near participant homes to support those with limited mobility or in areas with transportation or safety barriers. Future implementation should apply the same systematic approach to reducing referral barriers as was applied to enrollment and monitoring.

The 68.5% device return rate was higher than anticipated, but presents sustainability considerations for scale-up. The drivers of this return rate cannot be disentangled from the study design: the $10 USD reimbursement, the altruistic framing of return as benefiting fellow Hondurans, and the trust developed during the monitoring period likely all contributed, but their relative weights cannot be estimated from our data. This distinction matters for scale-up cost modeling, as a return mechanism reliant primarily on social trust may not survive program adaptation to another context, while one reliant primarily on financial incentives would require sustained budget allocation. Future studies should co-design return mechanisms in partnership with target populations and ensure independent measurement of these drivers through variation in incentive amount, messaging framing, and timing. For future implementation, programs should consider budgeting for expected replacement rates, establishing community-based collection points, or assigning devices to community health workers to distribute, collect, and maintain. As device costs decrease, single-use provision may eventually become more cost-effective than incentivizing device return. Cost-effectiveness analysis comparing device costs, transportation barriers, staff time, and logistics against clinical benefits should guide decisions.

Additionally, the staffing model warrants careful consideration for scale-up. The most clinically skilled task in this intervention is daily monitoring, which requires real-time recognition of warning signs and referral judgment; this is appropriately delivered by nurses or clinicians. Enrollment, by contrast, is largely procedural and could be safely task-shifted to trained, supervised personnel. Reallocating staff in this way could free clinicians for the monitoring role, extend program reach to a larger population, and potentially reduce facility burden by providing reassurance to the worried well who might otherwise present for care. The high fidelity we achieved depended in part on dedicated study staff; sustaining this in routine practice will require explicit attention to matching task complexity to provider skill level.

### Strengths and limitations

This study’s strengths include participatory design with multi-disciplinary stakeholder input, iterative and adaptive implementation allowing real-time problem-solving, and triangulation across multiple data sources (trial data, acceptability assessments, provider interviews, implementation documentation).

Discussions with key stakeholders and study staff led to substantive changes to intervention and implementation design. While the use of human-centered design was not an explicit goal, placing participants at the center, speaking with members of the target population, anticipating challenges they might face, and prioritizing accessible participation reflect that approach.

However, there are important limitations. The parent trial was not designed as a fidelity study, so results are observational. Reliance on self-reported measures for key outcomes (pulse oximeter use, satisfaction, warning sign reporting) introduces potential social desirability bias, as study staff administered assessments and participants may have provided courtesy responses. SESAL-employed physicians were aware that the study required phone access, and may have pre-screened participants informally, despite study guidelines, potentially introducing unmeasured selection bias. Quality of delivery was assessed subjectively, without consistent application of a validated framework, which could introduce recall and familiarity bias. Referral follow-through may be misclassified due to incomplete documentation systems and reliance on participant self-report, meaning actual adherence rates could differ from reported rates. Knowledge about COVID-19, silent hypoxia, and pulse oximetry evolved during the trial, creating challenges in balancing new insights with maintaining protocol fidelity. In addition, study physicians were stationed at triage centers during a 12-h daily window; patients presenting outside those hours could not be invited to participate, which may have introduced selection bias if their characteristics or risk profile differed systematically from those enrolled during staffed hours. Because participant-level WhatsApp engagement was not measured, we cannot isolate its independent contribution to fidelity outcomes, and we acknowledge this as an uncontrolled adaptation of the intervention. Family member involvement was not measured at the participant level, so its independent contribution to fidelity outcomes cannot be estimated. Finally, the study was conducted exclusively in urban Tegucigalpa and Comayagüela, where infrastructure differs substantially from rural Honduras; generalizability to rural settings should not be assumed.

Future research on remote patient monitoring and self-administered pulse oximetry should prioritize hybrid effectiveness-implementation trials that test both interventions and implementation strategies simultaneously. Before deploying a self-guided approach for follow-up care, researchers should engage with target populations to understand physical, social, and economic barriers, then develop and measure strategies to address those barriers to promote access and equity.

Application-based monitoring approaches warrant attention as a potential way to reduce human resource demands, although their fidelity, equitable reach, and acceptability are likely to vary across populations. During study design, we explored an application-based approach but were advised by SESAL personnel that this would be inappropriate for the target population. Beyond access, the high fidelity we observed appeared to depend heavily on the trust and rapport built between study nurses and participants during daily calls, a relational dimension that an application might not replicate. Hybrid approaches that combine application-based check-ins for digitally connected participants with nurse-delivered telephone monitoring for older or less connected populations may offer a useful compromise between efficiency, equity, and the relational quality of care.

Future studies should also expand the fidelity measurement framework to capture variables that this study could not. Digital messaging engagement and family member involvement should be measured directly as fidelity-relevant variables, particularly in populations where individual technology access is uneven but household support is robust. Mental health outcomes should be measured prospectively using validated instruments, given that social isolation is inherent to acute infectious illness and that the daily monitoring call appears to provide meaningful relational support. This may identify a substantial secondary benefit of remote monitoring with implications for program design, valuation, and prioritization beyond direct clinical outcomes.

This approach has broad applications for healthcare systems in Honduras and beyond, particularly during health emergencies. Our success with remote monitoring in a setting previously unfamiliar with telehealth demonstrates the potential for scaling telehealth solutions in both emergency and primary care contexts. As the Pan American Health Organization prioritizes digital health transformation, we must focus on how telehealth can improve access for marginalized populations and be leveraged during outbreaks and other public health emergencies (56). However, as demonstrated, accounting for technology access and incorporating the support available within multigenerational households and community networks can contribute to feasibility. These interventions brought healthcare closer to patients and provided guidance on seeking care during acute respiratory illness. Such remote monitoring can help overcome access barriers during emergencies and among marginalized groups facing barriers related to limited mobility, transportation costs, or mistrust of traditional healthcare settings.

## Conclusion

This investigation examined the implementation fidelity of remote patient monitoring and self-administered pulse oximetry in urban Tegucigalpa and Comayagüela, Honduras, during the COVID-19 health emergency. The intervention achieved high fidelity through context-appropriate implementation strategies developed with local expertise and multidisciplinary input, challenging assumptions that technology barriers prohibit such interventions in LMIC. However, critical implementation gaps emerged in referral adherence and device return rates, and substantial age-related disparities in phone access raise equity concerns for scale-up. These findings demonstrate that while remote patient monitoring can be delivered with high fidelity in well-resourced urban contexts, successful implementation requires: proactive strategies to ensure equitable technology access, health system integration to address referral barriers, and sustainability mechanisms to manage device supply. Future research should prioritize hybrid effectiveness-implementation trials that test interventions and strategies simultaneously, with explicit attention to generalizability, scalability, equity, and accessibility for marginalized populations. With appropriate implementation support and attention to context-specific barriers, these approaches have potential to enhance healthcare access and support digital health transformation during health emergencies and routine care in LMICs and beyond.

## Data Availability

Existing datasets are available in a publicly accessible repository: Publicly available datasets were analyzed in this study. This data can be found here: https://dataverse.harvard.edu/dataverse/honduraspulseoximetry/.
