# Supplementary material for: Improving fidelity of implementation of self-administered pulse oximetry and remote patient monitoring in Honduras during the COVID-19 pandemic
Source: Front Med (Lausanne). 2026 Jul 1;13:1721063. doi: 10.3389/fmed.2026.1721063 (PMC13370803; doi:10.3389/fmed.2026.1721063)
Supplement: Supplementary file 1 [file Data_Sheet_1.docx]

**Instrucciones para registrar sus datos:**

***Si su nivel de SpO_2_% está establemente por debajo del 92%, llame al equipo del estudio y / o regrese al centro de triaje de inmediato.***

Use el oxímetro de pulso para ver su nivel de saturación de oxígeno (SpO_2_%) todos los días en los tres momentos especificados:

1. En la mañana
2. En la noche
3. Durante la llamada de evaluación con la enfermera del estudio.

Use la tabla abajo para registrar su SpO_2_%. Recuerda que debe registrar el número más bajo y estable de un periodo de monitoreo de un minuto. Si el oxímetro de pulso no funciona alguna vez, anótalo. Si se olvide medir su nivel de saturación de oxígeno alguna vez, marca --

| **Día** | **Mañana**  Cuando se levante | **Noche**  Antes de dormir | **Llamada de evaluación** |
| --- | --- | --- | --- |
| 1 Día de diagnosis |  |  |  |
| 2 |  |  |  |
| 3 |  |  |  |
| 4 |  |  |  |
| 5 |  |  |  |
| 6 |  |  |  |
| 7 |  |  |  |
| 8 |  |  |  |
| 9 |  |  |  |
| 10 |  |  |  |

**Instrucciones para usar el oxímetro de pulso:**

¿Qué es un oxímetro de pulso?

• Un pequeño dispositivo que se usa para monitorear los niveles de oxígeno en la sangre.

• Se coloca en la punta de un dedo y el dispositivo utiliza rayos de luz para medir indirectamente el nivel de oxígeno en la sangre sin tener que extraer una muestra de sangre.

**Nivel de saturación de oxigeno**

**
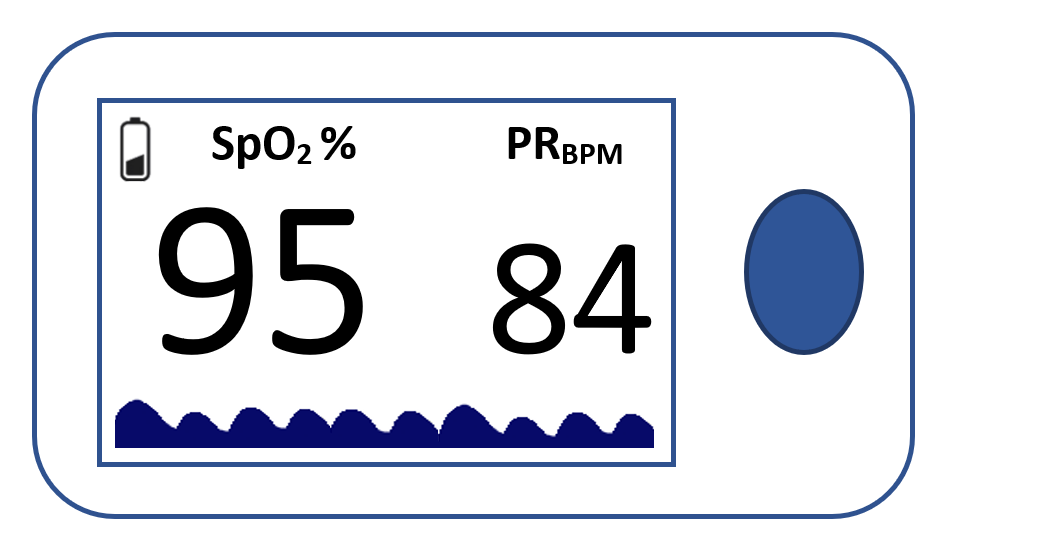
**

**Pulso**

**Batería**

**Onda del pulso**

**Botón de encendido**

**Instrucciones:**

1. Coloque su dedo en el dispositivo.
2. Presione el botón de encendido para prender el dispositivo.

(El dispositivo se apagará automáticamente después de 8 segundos si no detecta un dedo, solo tiene que presionar el botón de encendido de nuevo para prenderlo)

1. Siéntese quieto y no mueva su mano mientras se toma la lectura.
2. Vea los datos en la pantalla del dispositivo.
3. Después de un minuto, anote el nivel de oxígeno (SpO_2_%) en la tabla al otro lado del papel.

* Puede ser que los datos fluctúan durante la lectura. Se tiene que registrar **el nivel más bajo y estable** (sin fluctuaciones sustanciales) que vea durante el periodo de monitoreo de un minuto.


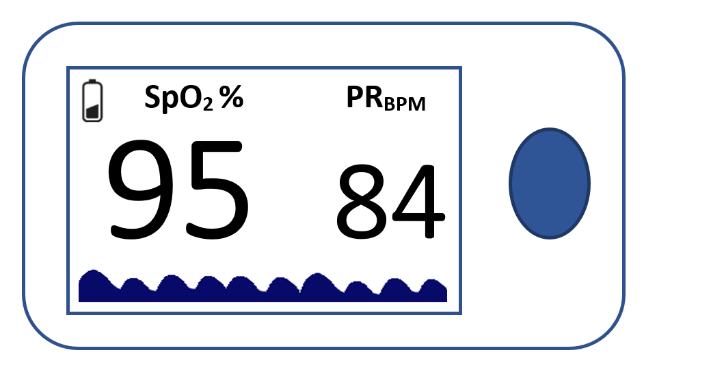


**?**

* Si la pantalla muestra “**?**”, significa que la señal es inestable. Mantenga las manos quietas y relajadas. Vuelva a intentarlo.
