# Supplementary material for: Improving fidelity of implementation of self-administered pulse oximetry and remote patient monitoring in Honduras during the COVID-19 pandemic
Source: Front Med (Lausanne). 2026 Jul 1;13:1721063. doi: 10.3389/fmed.2026.1721063 (PMC13370803; doi:10.3389/fmed.2026.1721063)
Supplement: Supplementary file 2 [file Data_Sheet_2.pdf]

S2 – Parent Trial Profile

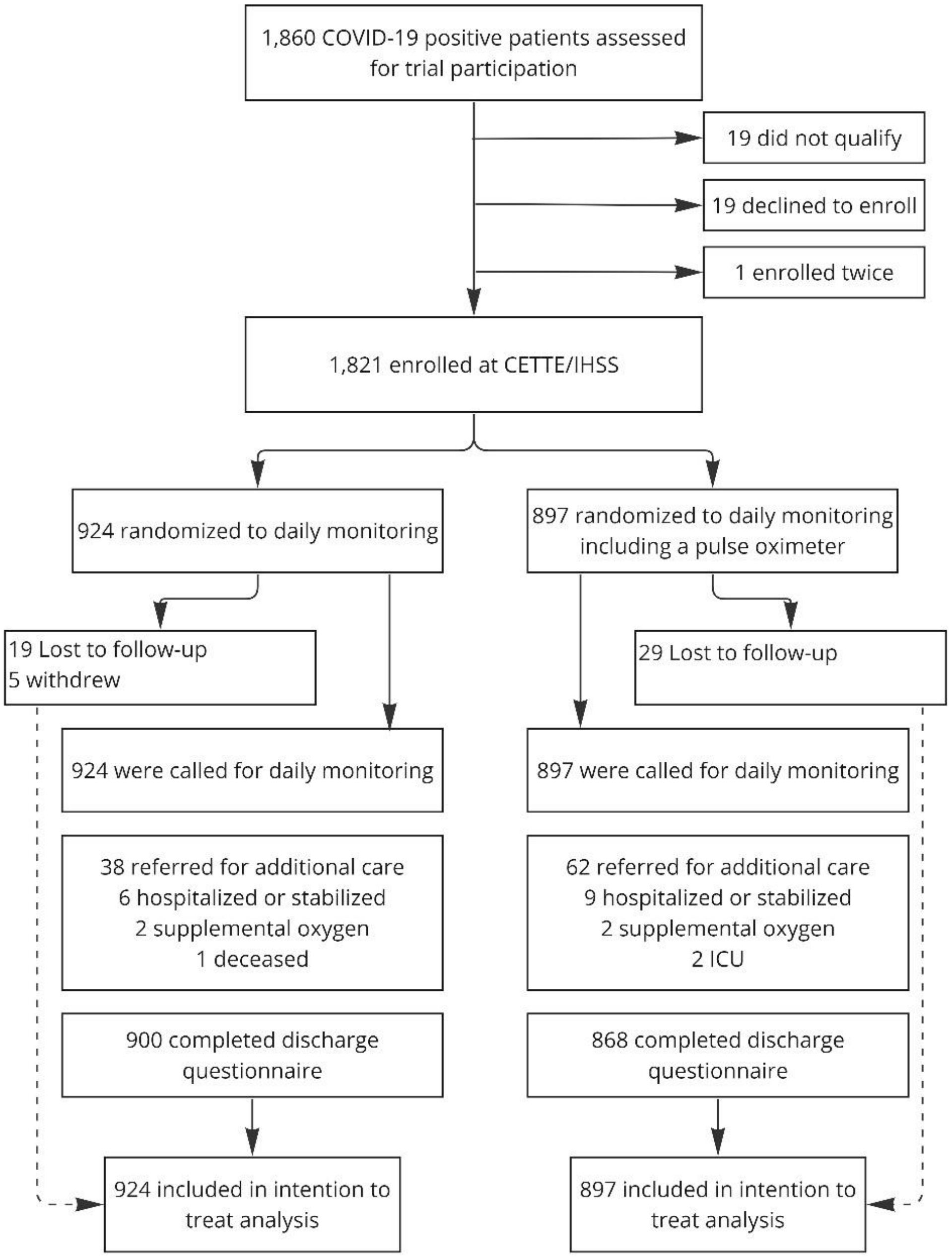

Reprinted with permission from Roberts et al., licensed under CC0 1.0:

Roberts KW, Alvarez B, de St Aubin M, Diaz O, Garnier S, Schnorr CD, Cruz S, Pavon L, Ochoa A, See R, Medice S, Santos HM, Ochoa J, Solis S, Dumas D, Baldwin M, Martinez A, Hakim A, Nilles E. Impact of self-administered pulse oximetry among non-hospitalized patients at risk of severe COVID-19 in Honduras: A pragmatic, cluster-randomized trial with temporal clustering. PLOS Glob Public Health. 2025 Nov 7;5(11):e0004618. doi: 10.1371/journal.pgph.0004618. PMID: 41202024; PMCID: PMC12594330.
